# The Effects of Aromatherapy on Premenstrual Syndrome Symptoms: A Systematic Review and Meta-Analysis of Randomized Clinical Trials

**DOI:** 10.1155/2020/6667078

**Published:** 2020-12-21

**Authors:** Somayeh Es-haghee, Fatemeh Shabani, Jessie Hawkins, Mohammad Ali Zareian, Fatemeh Nejatbakhsh, Marzieh Qaraaty, Malihe Tabarrai

**Affiliations:** ^1^Department of Traditional Medicine, School of Persian Medicine, Tehran University of Medical Sciences (TUMS), Tehran, Iran; ^2^Department of Midwifery, School of Medicine, Arak University of Medical Sciences, Arak, Iran; ^3^Integrative Health, Franklin School of Integrative Health Sciences, Franklin, TN, USA; ^4^Research Development Unit (CRDU), Sayad Shirazi Hospital Golestan University of Medical Sciences, Gorgan, Iran

## Abstract

*Objectives*Premenstrual syndrome (PMS) is a common disturbance among women of childbearing age. Aromatherapy is a commonly used form of complementary and alternative medicine (CAM) to treat PMS. The purpose of this study is to quantify and summarize the effects of aromatherapy on premenstrual syndrome symptoms. *Methods*. PubMed, Scopus, and Cochrane Library databases were searched through relevant search terms until October 2020. The effect sizes were pooled as weighted mean difference (WMD) and 95% confidence interval (CI) using the random effect model. Egger tests and visual inspection of the funnel plot were performed to identify the existence of publication bias. The *I*-squared (*I*^2^) test was applied to measure heterogeneity. *Results*. Eight studies (*n* = 8) were included in this analysis. The quantitative synthesis of evidence found that aromatherapy decreases PMS scores (WMD –13.83; 95% CI (−22.04, −5.63), *I*^2^ = 94.5%), total psychological symptoms of PMS (WMD –3.51; 95% CI (−4.84, −2.18), *I*^2^ = 82.6%), anxiety of PMS (WMD–1.78; 95% CI (−3.17, −0.38), *I*^2^ = 94.2%), depression of PMS (WMD–2.0; 95% CI (−3.65, −0.34), *I*^2^ = 93.7%), and fatigue of PMS (WMD – 1.44; 95% CI (−2.44, −0.44), *I*^2^ = 89.7%) compared to the control group. *Conclusion*. Aromatherapy is an effective tool for the relief of PMS symptoms. Additional randomized controlled clinical trials with different durations and essential oils should be conducted to confirm our findings.

## 1. Introduction

Premenstrual syndrome (PMS) refers to unpleasant changes in psychological, physical, and behavioral health that occur in the last week of the menstrual cycle and resolves at the beginning of the new menstrual cycle [1]. This syndrome can be quantified by the sum of psychological symptoms (anxiety/tension, depression, confusion, anger/irritability, mood swings, vigor, fear of rejection, lethargy, and sleep disorders), as well as physical symptoms (tenderness of the breasts, bloating, appetite changes, weight gain, headache, aches, abdominal pain, swelling, fatigue, gastrointestinal symptoms, and skin problems) [2]. Although the complete etiology of PMS is unclear, it can be partly attributed to hormonal changes during the menstrual cycle and the subsequent effect on neurotransmitters such as gamma-aminobutyric acid (GABA) and serotonin [2, 3].

PMS affects 20% to 40% of women of childbearing age all over the world [2, 3]. Because PMS can disrupt both the professional and personal lives of women [4], safe and effective treatments are urgently needed. Some medications such as psychotropic medications (e.g., selective serotonin reuptake inhibitors), hormone treatments (estradiol and progesterone), or nonsteroidal anti-inflammatory drugs (NSAIDs) have been prescribed for treatment of PMS [3]. Due to the side effects and lack of therapeutic response in some patients, women have turned to other therapeutic approaches [5]. Complementary and alternative medicine (CAM) is widely used as a safe, lower cost, alternative solution for coping with common health concerns such as PMS [6, 7].

Aromatherapy is a component of CAM which involves the inhalation of plant extracts as essential oils [8]. Essential or volatile oils are said to stimulate olfactory receptor cells and consequently send messages to the limbic system, the emotional center of the brain [9]. Previous meta-analyses have documented the beneficial effects of aromatherapy for dysmenorrhea [10], depressive symptoms [11], and sleep improvement [12].

One previous review evaluated the effectiveness of aromatherapy in conjunction with Iranian herbal medicines on PMS and primary dysmenorrhea [13]. However, this review focused on Iranian countries and omitted many trials conducted elsewhere in the world. Furthermore, the review focused on primary dysmenorrhea rather than PMS and did not conduct quantitative meta-analysis on PMS specifically. The existing scientific literature includes multiple randomized clinical trials evaluating the effects of aromatherapy on PMS. Some of them identified beneficial effects of aromatherapy on PMS symptoms [14–16]. To our knowledge, there is no meta-analysis evaluating the entirety of the clinical literature on the effects of aromatherapy on PMS symptoms. The purpose of this study is to quantify and summarize the findings of RCTs regarding the effects of aromatherapy on premenstrual syndrome.

## 2. Method

### 2.1. Search Strategy

This study was designed according to Preferred Reporting Items for Systematic Reviews and Meta-Analyses (PRISMA) [17].

Databases such as PubMed/Medline, Cochrane Library, and Scopus were searched to recognize relevant published articles until October 2020. The advanced search was performed using a prepared syntax based on Medical Subject Headings (MeSH) and related keywords including (“Aroma” OR “Aromatherapy” OR “Aromatic therapy” OR “Essential oil” OR “Fragrance” OR “Fragrant oil” OR “Scent”) AND (“Premenstrual Syndrome” OR “Premenstrual Tension” OR “Premenstrual Dysphoria” OR “Premenstrual Dysphoric Disorder”). To identify all possible studies, the search was conducted without the use of filters. In addition, the reference lists from eligible papers and pertaining review articles were manually inspected to ensure no other articles were missed.

### 2.2. Eligibility Criteria

Based on the predefined eligibility criteria, two independent investigators (SE and MQ) reviewed the title and abstract of every article for possible inclusion in this study. The inclusion criteria were (1) randomized clinical trials (RCTs) with either parallel or crossover design; (2) studies conducted on females with premenstrual syndrome; (3) studies assessing the effect of aromatherapy on premenstrual symptoms; and (4) studies reporting mean and standard deviation (SD) of symptoms of PMS. Studies which used other methods in addition to aromatherapy were omitted unless the control group also received the additional treatment. This was done to ensure that the aromatherapy treatment was the only difference between the groups.

### 2.3. Data Extraction

Data extraction was conducted by two separate authors who thoroughly searched each eligible article to extract the following data: author's name, publication year, country, essential oil selection, control group intervention, duration of intervention, duration of the treatment session, the total number of sessions, participants mean age or age range, sample size, study design, outcome assessment tool, and PMS symptom values (mean and standard deviation) before and after the intervention.

### 2.4. Risk of Bias Assessment

The risk of bias for each study was assessed by two independent examiners using the Cochrane Collaboration tool [18]. This scale evaluates six items: random sequence generation, allocation concealment, blinding (patients, personnel, and outcome assessors), incomplete outcome data, selective reporting, and other sources of bias. Studies were ranked as low risk, ambiguous risk, or high risk of bias based on each item.

### 2.5. Statistical Method

Data were reported as weighted mean differences (WMDs) with 95% confidence intervals (CIs). The random effects model was used to assess the weighted mean difference between values of PMS symptoms. For studies which did not provide mean change with standard deviations, we calculated these data using the following formula: mean change = final values − baseline values; SD = square root ((SD baseline)^2^ + (SD final)^2^ – (2*R* × SD baseline × SD final)) [19]. A correlation coefficient equal to 0.9 was used for the *R* value in the abovementioned formula [19, 20]. To convert standard deviations (SDs) to standard errors (SEs), we used the following formula: SD = SEM × sqrt (*n*), where *n* is the number of participants.

Heterogeneity was evaluated using the *I*^2^ index, and *I*^2^ values >50% were considered to be evidence of heterogeneity. Subgroup analyses were conducted based on predefined factors, including sample size, duration of intervention, outcome assessment tool, and study design. Sensitivity analyses were performed to examine the influence of each study on the overall effect size. Potential publication bias was identified by Egger's test and a visual inspection of funnel plots. In the presence of publication bias, Duval and Tweedie's trim and fill method was used to control the analysis for its effects [21]. All statistical analyses were conducted via STATA (Version 12.0, Stata Corp, College Station, TX). Statistical significance was defined as a *P* value below 0.05.

## 3. Result

**Figure 1:**
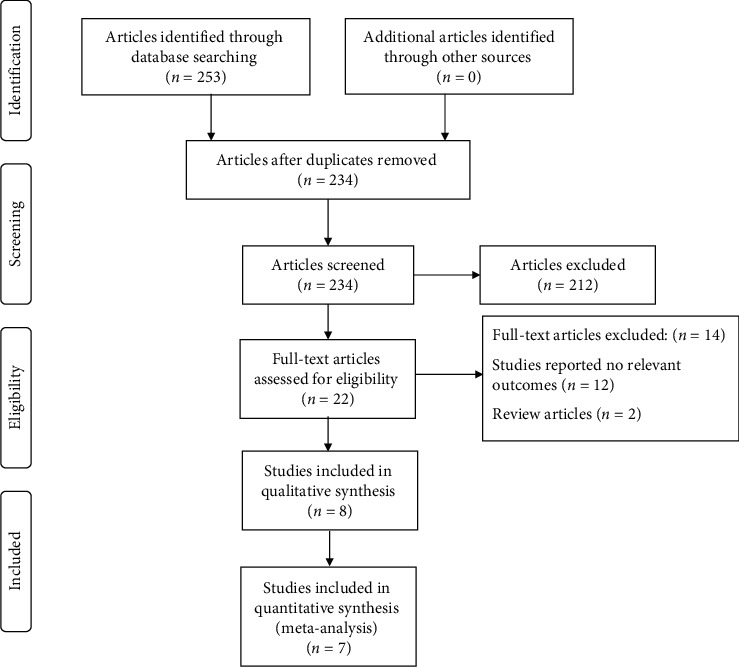
Flow diagram of study selection.

A total of 253 articles were identified via online databases, and no additional articles were found through the additional manual search. A total of 234 articles remained after the elimination of duplicates. The abstracts of these 234 papers were screened, and the full text of 22 studies was evaluated. Of those 22 studies, 14 articles were omitted for the following reasons: 12 studies reported irrelevant outcomes, and 2 studies were review articles. This left a total of 8 studies in this analysis. The flow diagram of the study selection process is shown in Figure 1.  Table 1 presents details of eligible studies. These studies represented a total of 295 participants and were published between 2016 and 2020. Six of the studies had parallel design, and the other 2 studies had a crossover design. Studies were conducted in Iran [15, 16, 22, 23], Japan [24, 25], India [26], and Turkey [14]. All of the studies were published in the English language.

Study participants were women who had moderate to severe PMS. Four of the studies measured PMS symptoms with PSST questionnaires [15, 22, 23, 26], two used PMOS [24, 25], one used ACOG [14], and one used the PMS score [16] questionnaire. One of the studies used aromatherapy with massage while the other seven studies used aromatherapy as the exclusive intervention [16]. Aromatherapy treatment time varied from 5 to 35 minutes, and total sessions ranged from 1 to 5. All of the studies used single oil as the intervention. Lavender was used in two studies [14, 25], as was *Citrus aurantium* blossom [15, 23] and rose (*n* = 2) [22, 23]. Yuzu, a Japanese citrus fruit (*Citrus junos Sieb. ex Tanaka*) [24], geranium [16], and clary sage [26] were each used in one study. Three studies used a diffuser to administer the aromatherapy treatment [24–26], three studies used eye pad [15, 22, 23], one study used steam inhalation [14], and one study used massage [16] as the method of administration.

### 3.1. Assessment of the Risk of Bias

Five studies were categorized as high quality, and the remaining articles were classified as fair, based on six domains of the Cochrane Collaboration tool. Only three studies described the exact method used for randomization, and only two studies reported blinding. The details of quality assessment for articles included in the present systematic review are illustrated in Table 2.

### 3.2. Meta-Analysis

#### 3.2.1. The Effect of Aromatherapy on Psychological Symptoms of PMS

**Figure 2:**
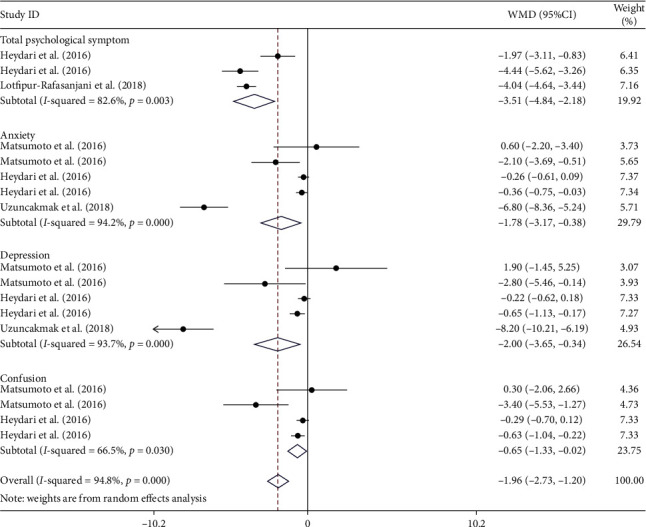
Forest plot showing the effects of aromatherapy on psychological symptoms of PMS (WMDs and 95% CIs) in women with PMS using the random effects model. CI, confidence interval; PMS, premenstrual syndrome; WMD, weighted mean difference.

Seven of the studies examined the impact of aromatherapy on psychological symptoms of PMS [14–16, 22–25]. This meta-analysis found that aromatherapy treatment decreases psychological symptoms of PMS (WMD–3.51; 95% CI (−4.84, −2.18), *I*^2^ = 82.6%), anxiety of PMS (WMD–1.78; 95% CI (−3.17, −0.38), *I*^2^ = 94.2%), and depression of PMS (WMD–2.0; 95% CI (−3.65, −0.34), *I*^2^ = 93.7%) in the intervention group compared to the control group. However, aromatherapy did not produce a significant effect on confusion as a symptom of PMS (WMD–0.65; 95% CI (−1.33, 0.02), *I*^2^ = 66.5%) (Figure 2).

#### 3.2.2. The Effect of Aromatherapy on Physical Symptoms of PMS

**Figure 3:**
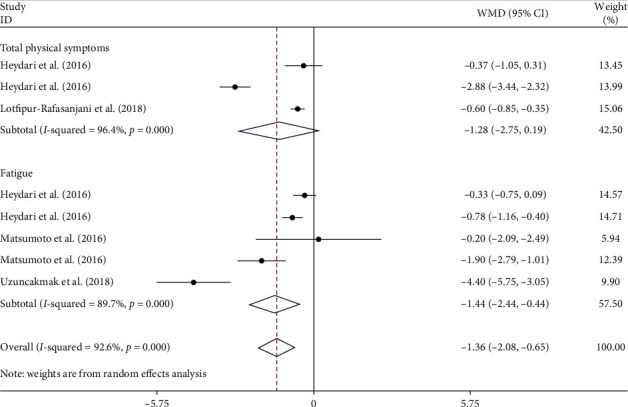
Forest plot showing the effects of aromatherapy on physical symptoms of PMS (WMDs and 95% CIs) in women with PMS using the random effects model. CI, confidence interval; PMS, premenstrual syndrome; WMD, weighted mean difference.

All eight studies investigated the impact of aromatherapy treatment on physical symptoms of PMS [14–16, 22–26]. This meta-analysis found that aromatherapy treatment significantly reduces physical symptoms of PMS (WMD–1.28; 95% CI (−2.75, 0.19), *I*^2^ = 94.6%) as well as fatigue from PMS (WMD–1.44; 95% CI (−2.44, −0.44), *I*^2^ = 89.7%) based on the random effects model (Figure 3).

#### 3.2.3. The Effect of Aromatherapy on the Overall Score of PMS

**Figure 4:**
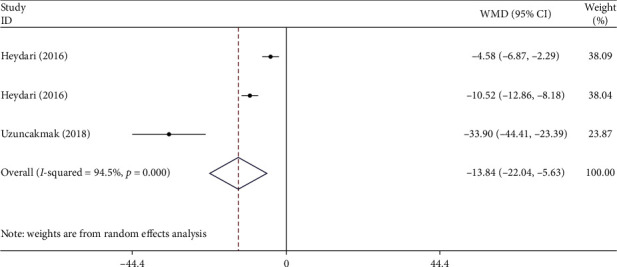
Forest plot showing the effects of aromatherapy on total score of PMS (WMDs and 95% CIs) in women with PMS using the random effects model. CI, confidence interval; PMS, premenstrual syndrome; WMD, weighted mean difference.

Three of the studies examined the impact of aromatherapy on the overall score of PMS [14, 15, 22]. This meta-analysis found that aromatherapy also decreased the severity of PMS in the intervention group compared to the control group (WMD–13.83; 95% CI (−22.04, −5.63), *I*^2^ = 94.5%) (Figure 4).

#### 3.2.4. Subgroup Analyses

Subgroup analyses were conducted based on predefined factors such as symptom, sample size, duration of treatment, study design, and outcome assessment tool. The duration of treatment and outcome assessment tools were identified as the sources of heterogeneity regarding the psychological symptoms of PMS. However, none of these factors were found to be sources of heterogeneity regarding the physical symptoms of PMS. Furthermore, aromatherapy had a more favorable effect on psychological symptoms of PMS and physical symptoms of PMS in studies with a crossover design, studies which used the ACOG questionnaire for PMS measurement, studies with higher duration, and studies with smaller sample sizes (*P* < 0.001 for all) (Table 3).

#### 3.2.5. Sensitivity Analyses

By omitting each study and reanalyzing the data, we found that none of the studies affect the significance of the summary effect size of aromatherapy on psychological symptoms of PMS (Supplementary Figure 1), physical symptoms of PMS (Supplementary Figure 2), or the overall score of PMS (Supplementary Figure 3).

### 3.3. Publication Bias

The funnel plot and Egger test (*P*=0.039) identified publication bias regarding the effect of aromatherapy on psychological symptoms of PMS (Supplementary Figure 4). Therefore, we performed Tweedie's trim and fill to adjust for this bias. This did not change the effect size. The funnel plot visually showed that there was no publication bias regarding physical symptoms of PMS (Supplementary Figure 5) and the overall score of PMS (Supplementary Figure 6). In addition, the Egger test also confirmed the findings regarding physical symptoms of PMS (*P*=0.22) and the overall score of PMS (*P*=0.38).

## 4. Discussion

This meta-analysis investigated the effect of aromatherapy on premenstrual syndrome symptoms in women. This study provides evidence that inhaling essential oils can alleviate the symptoms of PMS whether measured as psychological symptoms, physical symptoms, or total PMS symptom scores.

Psychological symptoms of PMS may occur following the decrease in quantity or function of serotonin, tryptophan, and estrogen level [27, 28]. A prospective, randomized controlled trial conducted on elderly persons with symptoms of depression showed an increase in 5-hydroxytryptamine (serotonin) concentrations in the aromatherapy group (lavender + sweet orange (*Citrus sinensis*) + bergamot (*Citrus bergamia*)) compared to the control group [29]. In a study conducted by Choi et al, daily inhalation of Citrus aurantium essential oil at concentrations of 0.5% for 5 days by postmenopausal women could increase the estrogen level slightly [30].

The present study found that aromatherapy could decrease the severity of psychological symptoms of PMS such as anxiety and depression, as well as fatigue as a physical symptom of PMS and the total score of PMS. Systematic review sand meta-analyses have found that aromatherapy can decrease depressive symptoms [11] and preoperative anxiety [31] in other demographic groups, which is consistent with our findings. In addition, one study found a favorable effect of aromatherapy with essential oils containing 0.5% neroli oil on ICU (intensive care unit) patients [30]. One clinical trial study showed that lavender aromatherapy could significantly decrease the mean scores of fatigue and anxiety of patients undergoing hemodialysis treatment [32]. However, one study with one session of aromatherapy with 5 min exposure did not find a significant effect on depressive symptoms on pregnant woman [33]. The insignificant result may be due to short intervention exposure time in the treatment groups.

### 4.1. Oil Selection

One important factor to consider when assessing the effectiveness of aromatherapy is that essential oils are plant extracts. Therefore, each batch differs with regard to the chemical volatile compounds contained within the oils used in these studies. The volatile compound of essential oils contains flavonoid and trepan chemicals such as limonene, gamma-terpinene, linalool, and linalyl acetate that have shown to act as anxiolytic antidepressants and contain sedative properties [34, 35]. Limonene, the main odorant of citrus fruits, plays a role in the stimulation of the sympathetic system and subjective alertness [36]. Linalool as a key volatile component of lavender has sedative properties through harnessing glutamate binding [37]. Gamma-Terpinene, another volatile compound found in yuzu, decreases stress by enhancing the dopamine release [38]. Another volatile component (*β*-caryophyllene) has been found to improve psychological symptoms such as depression and anxiety [39]. A major phytoncide, *α*-pinene, also has alleviating effects on autonomic stress response to novel environments [40]. Lehrner et al. showed after inhalation of fragrance from orange (*Citrus sinensis*), made up of limonene (88.1%), myrcene (3.77%), and *α*-pinene (1.19%), female patients had lower anxiety, higher peace, and a more positive mood in a waiting room of dental office [41].

One reason for the variation in previous clinical studies on aromatherapy might be the variation in distance between the aroma and the nostrils, which affects the overall dose of inhalation. Most studies in our analysis used a diffuser or eye pads, both of which are effective methods for producing favorable effects [42].

One explanation for aromatherapy's effects on PMS is the way in which these volatile compounds affect the limbic system. The volatile odorant molecules are inhaled through the roof of the nose where cilia enable them to reach the receptor cells in the nose. When these volatile odorant molecules reach these cells, the olfactory bulb and olfactory tract transmit an electrochemical impulse to the primary olfactory areas in the brain that are contained in the hypothalamus, hippocampus, and the limbic system. These systems are responsible for controlling autonomic homeostasis, managing conscious thought processes, and creating emotional feelings, respectively [37].

### 4.2. Limitations

The small number of studies that examined symptoms of PMS is a primary limitation of this study. Publication bias in the studies that examined the impact of aromatherapy on PMS scores produces another limitation although publication bias should be interpreted with caution given the small sample size. Furthermore, the heterogeneity among studies is high. The high amount of heterogeneity among studies may be due to the differences in duration of the treatment session, the total number of sessions, frequency of the treatment, forms of essential oils, different volatile compounds, and outcome assessment tool. In addition, three out of the 8 studies included in the systematic review were only found to be of fair quality.

## 5. Conclusion

The meta-analysis provides evidence that aromatherapy reduces overall symptom scores and both physical and psychological symptoms of PMS. To reproduce these results, a pretest is recommended before using aromatherapy, ensuring that participants have healthy olfactory function and do not experience negative responses to the oils selected. In addition, an increase in inhalation time and a higher number of sessions should be considered for future aromatherapy treatments.

## Figures and Tables

**Table 1:** Characteristics of randomized trials on the effects of aromatherapy on the premenstrual syndrome symptoms included in the meta-analysis.

Reference	Location	Publication year	Subjects and gender	Age, y^1^	Design	Intervention type		Duration (mo)	Outcome assessment tool	Outcomes	Findings	Notes about subjects
						Intervention	Control					
(1) Heydari et al.	Iran	2016	A : 33 C : 33	A : 22.48 ± 1.76 C : 21.84 ± 1.50	Parallel	*Citrus aurantium* blossom essential oil (0.5%) (eye pad)	Odorless sweet almond oil	2 (twice a day for 5 minutes for 5 days)	PSST	(i) Score of psychological symptoms (ii) Score of physical symptoms (iii) Score of social symptoms (iv) Score of PMS	Total score of PMS and psychological symptoms decreased significantly after aromatherapy compared to the control group; however, aromatherapy had not significant effects on physical and social symptoms	66 students with moderate PMS

(2) Heydari et al	Iran	2016	A : 33 C : 31	A:22.66 ± 3.41 C : 21.84 ± 1.50	Parallel	Essential oils of *Rosa damascena* (4%) (eye pad)	Aromatherapy with 100% sweet almond oil	2 (twice a day for 5 minutes for 5 days)	PSST	(i) Score of psychological symptoms (ii) Score of physical symptoms (iii) Score of social symptoms (iv) Score of PMS	Total score of PMS, psychological, physical, and social symptoms decreased significantly after aromatherapy compared to the control group	64 students with moderate PMS

(3) Heydari et al	Iran	2016	A : 33 C : 33	A:22.33 ± 2.38 C : 22.33 ± 2.38	—	*Citrus aurantium* blossom essential oil (0.5%) (eye pad) Essential oils of *Rosa damascena* (4%) (eye pad)	Sweet almond oil	2 (twice a day for 5 minutes for 5 days)	PSST	(i) Anxiety/tension (ii) Tearful/increased sensitivity to rejection (iii) Depressed mood/hopelessness (iv) Decreased interest in work activities (v) Decreased interest in home activities and decreased interest in social activities (vi) Difficulty in concentrating (vii) Feeling overwhelmed or out of control (viii) Fatigue/lack of energy (ix) Overeating/food craving (x) Insomnia (xi) Hypersomnia (xii) Physical symptoms: breast tenderness, headaches, joint/muscle pain, bloating, and weight gain (xiii) Symptom interference with work efficiency or productivity (xiv) Symptom interference with relationships with coworkers and friends (xv) Symptom interference with relationships with family (xvi) Symptom interference with social life activities (xvii) Symptom interference with home responsibilities	Psychological symptoms were significant between the three groups except for the variables of anxiety, interest in work activities, and social activities and insomnia; the aromatherapy with *Citrus aurantium* compared to placebo significantly reduced the score of symptoms such as tearful/increased sensitivity to rejection, feeling overwhelmed, or out of control; aromatherapy with *Rosa damascena* compared to placebo significantly reduced the anger/irritability, tearful/increased sensitivity to rejection, depression, hopelessness, difficulty in the concentration, and hypersomnia Rosa damascena had a significant effect on physical variables, such as fatigue and lack of energy, breast tenderness, headache, muscle and joint pain, bloating, and weight gain, but the effect of *Citrus aurantium* was not significant on any of the physical variables Aromatherapy with *Citrus aurantium* had a positive effect only on the relationship between the coworkers and friends, but *Rosa damascena* in addition to effect on this variable had a positive effect on family relationship, social life activities, and home responsibilities	66 students with moderate PMS

(4) Lotfipur-Rafsanjani et al.	Iran	2018	A : 37 C : 38	18–29 yrs	Parallel	Geranium 2% in almond oil + massage (30 min/week)	Sweet almond oil + massage (30 min/week)	2 (30 min/week)	PMS score	(i) Psychological symptoms (ii) Physical symptoms	Aromatherapy massage decreased the PMS physical and mental symptoms significantly compared to the massage therapy	75 students with PMS

(5) Matsumoto et al.	Japan	2016	A : 9 C : 8	20.6 ± 0.2	Crossover	Fragrance from yuzu, a Japanese citrus fruit (*Citrus junos* Sieb. ex Tanaka) (diffuser)	Lavender	1 (35 min)	POMS	(i) Tension and anxiety (ii) Depression and dejection (iii) Anger and hostility (iv) Vigor (v) Fatigue (vi) Confusion (vii) High-frequency power	Tension-anxiety, anger-hostility, and fatigue improved in the aromatherapy group compared to the control group; however, other symptoms did not change significantly	17 women with moderate PMS

(6) Matsumoto et al.	Japan	2016	A : 9 C : 8	21.7 ± 0.8	Crossover	Lavender (diffuser)	Water	1 (35 min)	POMS	(i) Tension and anxiety (ii) Depression and dejection (iii) Anger and hostility (iv) Vigor (v) Fatigue (vi) Confusion (vii) High-frequency power	Depression-dejection and confusion declined significantly in the aromatherapy group; however, other symptoms did not change significantly	17 women with moderate PMS

(7) Uzuncakmak et al.	Turkey	2018	A : 40 C : 37	—	Crossover	Lavender (steam inhalation)	—	3 (5 sessions on average for each cycle)	ACOG	(i) Anxiety (ii) Depressive effect (iii) Fatigue (iv) Nervousness (v) Pain (vi) Appetite change (vii) Sleep-related changes (viii) Swelling (ix) Depressive thought (x) PMS scale	Aromatherapy improved the PMS scale and subdimensions of anxiety, depressive affect, nervousness, pain, bloating, and depressive thought mean scores compared to the control group	87 students with PMS

(8) Geethanjali et al	India	2020	A : 30 C : 30	18–35	Parallel	Clary sage (Salvia sclerae) (diffuser)	Water	1 (20 min)	PSST	(i) High-frequency power	Aromatherapy increased high-frequency power significantly compared to the control group	60 women with PMS

^1^Values of overall ranges and mean ± SDs in each group. A, aromatherapy; ACOG, American College of Obstetricians and Gynecologists; C, control; CI, confidence interval; PMS, premenstrual syndrome; POMS, Profile of Mood State; PSST, premenstrual symptoms screening tool; WMD, weighted mean difference.

**Table 2:** Cochrane risk of bias assessment for randomized controlled trials on the effect of sesame consumption on diabetic indices in adults.

Reference	Random sequence generation	Allocation concealment	Blinding of participants, personnel, and outcome assessors	Incomplete outcome data	Selective outcome reporting	Other sources of bias
Heydari et al.	L	U	L	L	L	L
Heydari al.	L	L	L	L	L	L
Heydari et al.	L	U	U	L	L	L
Lotfipur-Rafsanjani et al.	L	U	H	L	L	L
Matsumoto et al.	L	U	U	L	L	L
Matsumoto et al.	L	U	U	L	L	L
Uzuncakmak et al.	L	L	H	L	L	L
Geethanjali et al.	L	L	H	U	L	L

H, high risk of bias; L, low risk of bias; U, unclear risk of bias.

**Table 3:** Pooled estimates of the effects of aromatherapy on premenstrual syndrome symptoms within different subgroups.

	Number of trials		WMD (95% CI)		*P* value		*P* heterogeneity		*I* ^2^ (%)	
	Psychological symptoms	Physical symptoms	Psychological symptoms	Physical symptoms	Psychological symptoms	Physical symptoms	Psychological symptoms	Physical symptoms	Psychological symptoms	Physical symptoms
Total	17	8	−1.79 (−2.54, −1.04)	−1.27 (−1.96, −0.58)	<0.001	<0.001	<0.001	<0.001	96.2	92.9
PMS symptoms										
Score of psychological symptoms	3	—	−3.73 (−4.22, −3.25)	—	<0.001	—	0.003	—	82.6	—
Anxiety	5	—	−0.51 (−0.77, −0.26)	—	<0.001	—	<0.001	—	94.2	—
Depression	5	—	−0.58 (−0.88, −0.28)	—	<0.001	—	<0.001	—-	93.7	—
Confusion	4	-	−0.50 (−0.78, −0.21)	—	0.001	—	0.03	—	66.5	—
Score of physical symptoms	—	3	—	−0.90 (−1.12, −0.69)	—	<0.001	—	<0.001	—	96.4
Fatigue	—	5	—	−0.82 (−1.08, −0.56)	—	<0.001	—	<0.001	—	89.7

Study design										
Parallel	9	6	−0.73 (−0.88, −0.57)	−0.84 (−1.01, −0.67)	<0.001	<0.001	<0.001	<0.001	95.6	94.3
Crossover	8	2	−3.59 (−4.33, −2.84)	−1.62 (−2.45, −0.79)	<0.001	<0.001	<0.001	0.094	89.9	64.4

Outcome assessment tool										
PSST	8	4	−0.49 (−0.65, −0.32)	−0.95 (−1.18, −0.71)	<0.001	<0.001	<0.001	<0.001	87.3	94.7
POMS	6	2	−1.45 (−2.38, −0.52)	−1.62 (−2.45, −0.79)	<0.001	0.005	0.025	0.094	61.2	64.4
ACOG	2	1	−7.32 (−8.55, −6.09)	−4.40 (−5.74, −3.05)	<0.001	<0.001	0.28	—	14.2	—
PMS score	1	1	−4.04 (−4.63, −3.44)	−0.60 (−0.84, −0.35)	<0.001	<0.001	—	—	—	—
Duration of treatment										
<2 mo	6	2	−1.45 (−2.38, −0.52)	−1.62 (−2.45, −0.79)	0.002	0.005	0.025	0.094	61.2	64.4
=2 mo	9	5	−0.73 (−0.88, −0.57)	−0.78 (−0.95, −0.61)	<0.001	<0.001	<0.001	<0.001	95.6	93.4
>2 mo	2	1	−7.32 (−8.55, −6.09)	−4.40 (−5.74, −3.05)	<0.001	<0.001	0.28	—	14.2	—

Sample size										
<20	6	2	−1.45 (−2.38, −0.52)	−1.62 (−2.45, −0.79)	0.002	<0.001	0.025	0.098	61.2	64.4
≥20	11	6	−0.83 (−0.99, −0.68)	−0.84 (−1.01, −0.67)	<0.001	<0.001	<0.001	<0.001	96.6	95.3

ACOG, American College of Obstetricians and Gynecologists; CI, confidence interval; PMS, premenstrual syndrome; POMS, Profile of Mood State; PSST, premenstrual symptoms screening tool; WMD, weighted mean difference.

## Data Availability

The data used to support the findings of this study are available from the corresponding author upon request.

## Supplementary Materials

Supplementary Materials Supplementary Figure 1: analysis of the influence of aromatherapy on psychological symptoms of PMS. CI, confidence interval; PMS, premenstrual syndrome. Supplementary Figure 2: analysis of the influence of aromatherapy on physical symptoms of PMS. CI, confidence interval; PMS, premenstrual syndrome. Supplementary Figure 3: analysis of the influence of aromatherapy on total score of PMS. CI, confidence interval; PMS, premenstrual syndrome. Supplementary Figure 4: funnel plot for assessing publication bias in the studies reporting the effects of aromatherapy on psychological symptoms of PMS. PMS, premenstrual syndrome; SE, standard error; WMD, weighted mean difference. Supplementary Figure 5: funnel plot for assessing publication bias in the studies reporting the effects of aromatherapy on physical symptoms of PMS. PMS, premenstrual syndrome; SE, standard error; WMD, weighted mean difference. Supplementary Figure 6: funnel plot for assessing publication bias in the studies reporting the effects of aromatherapy on total score of PMS. PMS, premenstrual syndrome; SE, standard error; WMD, weighted mean difference.
