# Supplementary material for: The Effects of Aromatherapy on Premenstrual Syndrome Symptoms: A Systematic Review and Meta-Analysis of Randomized Clinical Trials
Source: Evid Based Complement Alternat Med. 2020 Dec 21;2020:6667078. doi: 10.1155/2020/6667078 (PMC7769645; doi:10.1155/2020/6667078)
Supplement: Supplementary Materials — Supplementary Figure 1: analysis of the influence of aromatherapy on psychological symptoms of PMS. CI, confidence interval; PMS, premenstrual syndrome. Supplementary Figure 2: analysis of the influence of aromatherapy on physical symptoms of PMS. CI, confidence interval; PMS, premenstrual syndrome. Supplementary Figure 3: analysis of the influence of aromatherapy on total score of PMS. CI, confidence interval; PMS, premenstrual syndrome. Supplementary Figure 4: funnel plot for assessing publication bias in the studies reporting the effects of aromatherapy on psychological symptoms of PMS. PMS, premenstrual syndrome; SE, standard error; WMD, weighted mean difference. Supplementary Figure 5: funnel plot for assessing publication bias in the studies reporting the effects of aromatherapy on physical symptoms of PMS. PMS, premenstrual syndrome; SE, standard error; WMD, weighted mean difference. Supplementary Figure 6: funnel plot for assessing publication bias in the studies reporting the effects of aromatherapy on total score of PMS. PMS, premenstrual syndrome; SE, standard error; WMD, weighted mean difference. [file 6667078.f1.docx]

**Supplementary Figure 1:** Analysis of the influence of aromatherapy on psychological symptoms of PMS. CI, Confidence interval; PMS, Premenstrual syndrome

**Supplementary Figure 2:** Analysis of the influence of aromatherapy on physical symptoms of PMS. CI, confidence interval; PMS, Premenstrual syndrome

**Supplementary Figure 3:** Analysis of the influence of aromatherapy on total score of PMS. CI, confidence interval; PMS, Premenstrual syndrome


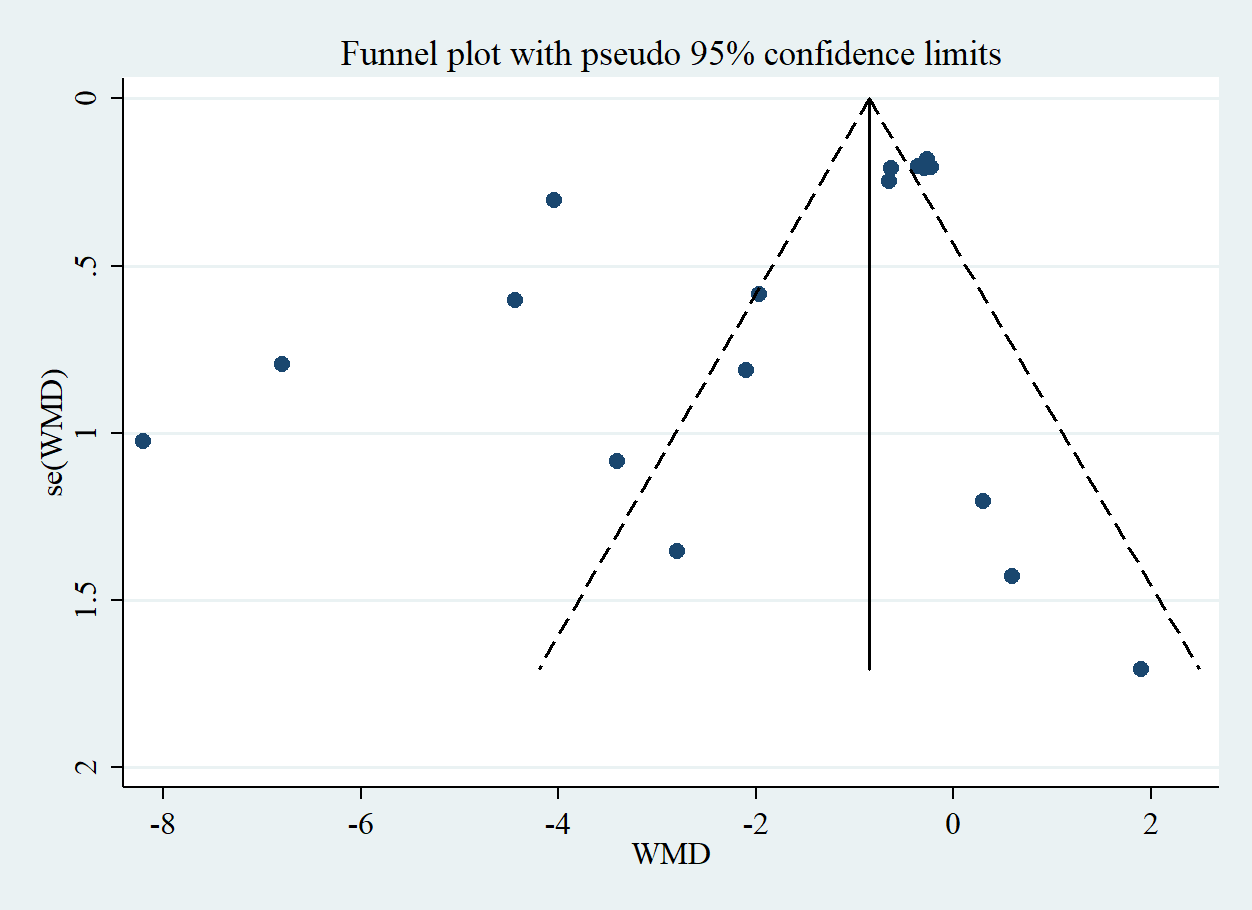


**Supplementary Figure 4:** Funnel plot for assessing publication bias in the studies reporting the effects of aromatherapy on psychological symptoms of PMS. PMS, Premenstrual syndrome; SE, standard error; WMD, weighted mean difference

**
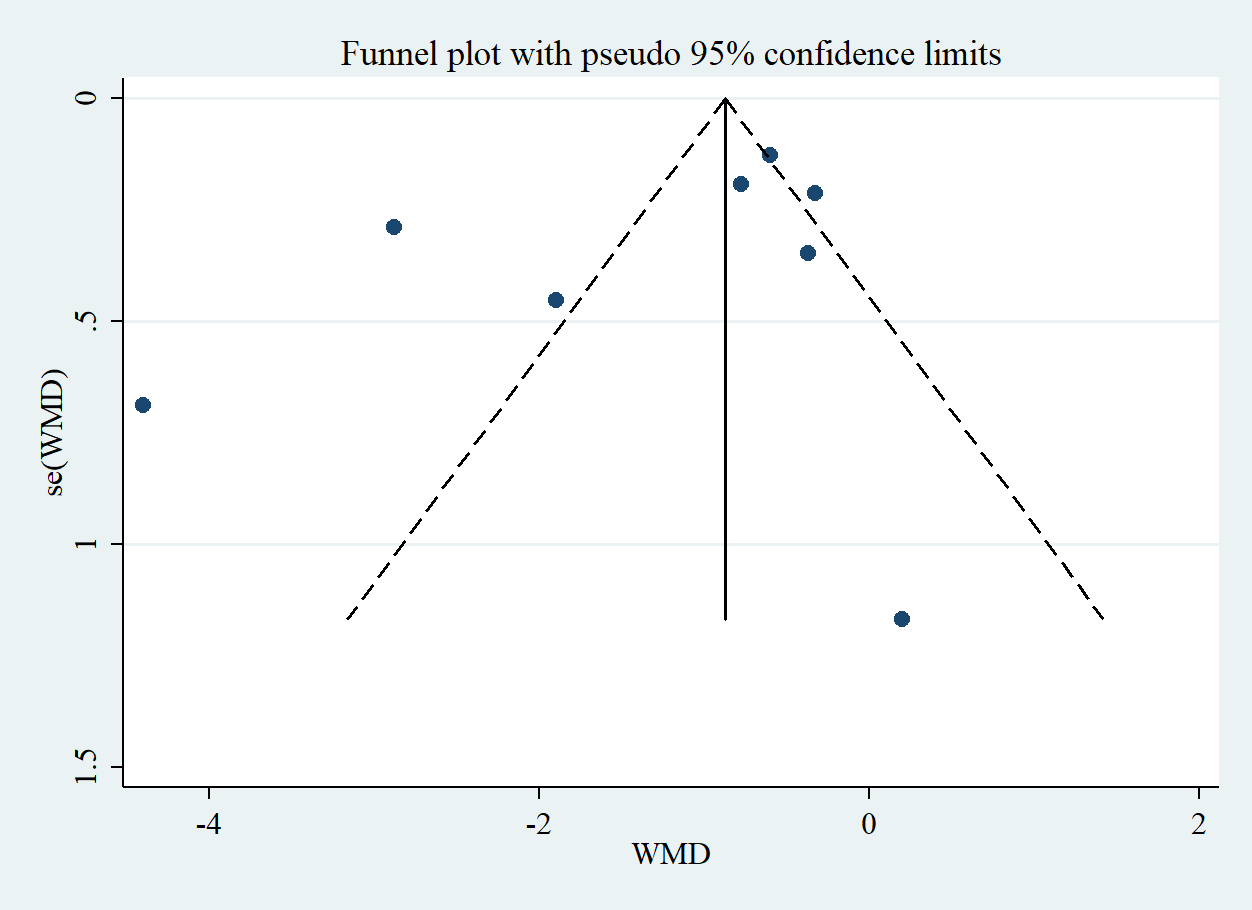
**

**Supplementary Figure 5:** Funnel plot for assessing publication bias in the studies reporting the effects of aromatherapy on physical symptoms of PMS. PMS, Premenstrual syndrome; SE, standard error; WMD, weighted mean difference


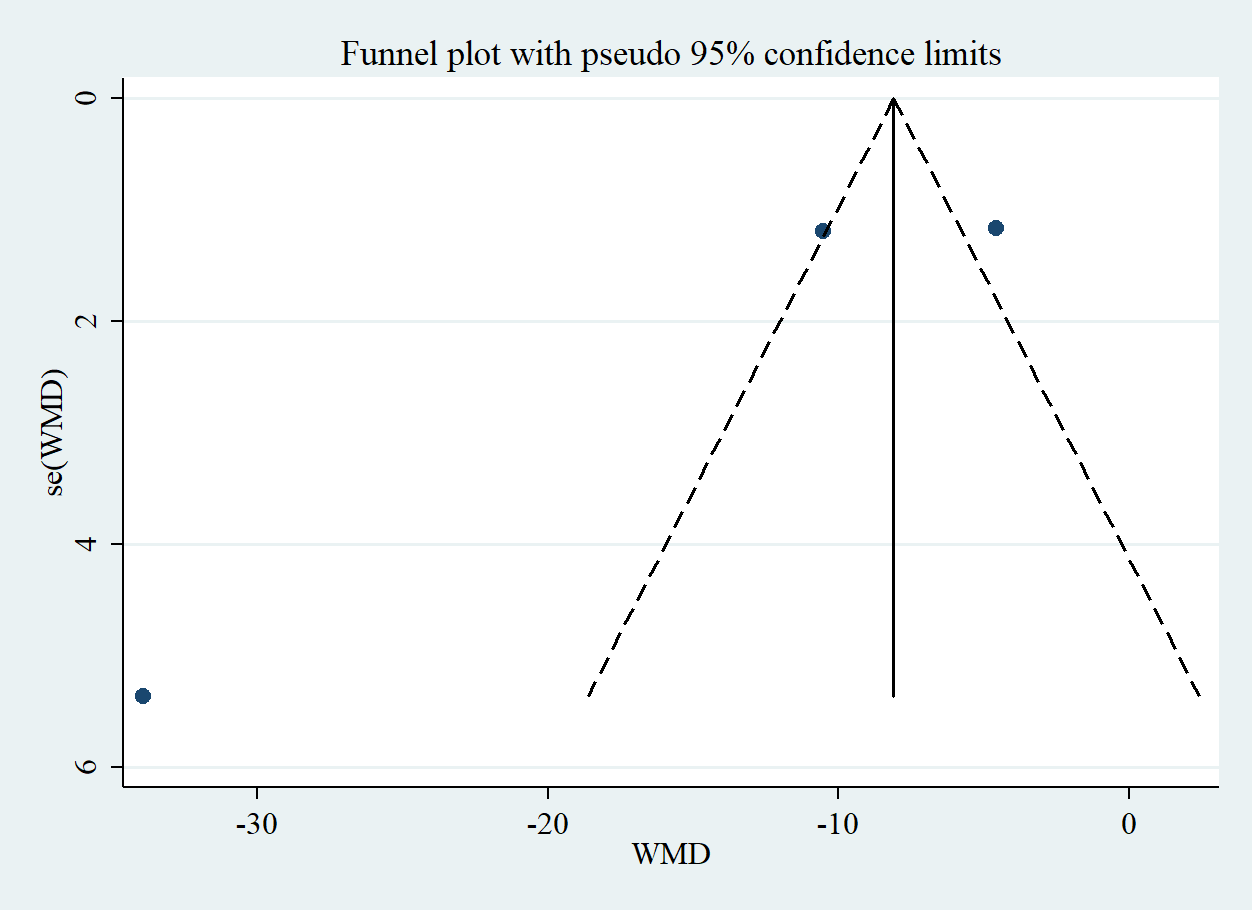


**Supplementary Figure 6:** Funnel plot for assessing publication bias in the studies reporting the effects of aromatherapy on total score of PMS. PMS, Premenstrual syndrome; SE, standard error; WMD, weighted mean difference
